# PCDH15 dual-AAV gene therapy for deafness and blindness in Usher syndrome type 1F models

**DOI:** 10.1172/JCI177700

**Published:** 2024-10-23

**Authors:** Maryna V. Ivanchenko, Daniel M. Hathaway, Eric M. Mulhall, Kevin T.A. Booth, Mantian Wang, Cole W. Peters, Alex J. Klein, Xinlan Chen, Yaqiao Li, Bence György, David P. Corey

**Affiliations:** 1 Department of Neurobiology, Harvard Medical School, Boston, Massachusetts, USA.; 2 Institute of Molecular and Clinical Ophthalmology Basel, Basel, Switzerland.; 3 Department of Ophthalmology, University of Basel, Basel, Switzerland.

**Keywords:** Ophthalmology, Otology, Gene therapy, Genetic diseases

## Abstract

Usher syndrome type 1F (USH1F), resulting from mutations in the protocadherin-15 (*PCDH15*) gene, is characterized by congenital lack of hearing and balance, and progressive blindness in the form of retinitis pigmentosa. In this study, we explore an approach for *USH1F* gene therapy, exceeding the single AAV packaging limit by employing a dual–adeno-associated virus (dual-AAV) strategy to deliver the full-length *PCDH15* coding sequence. We demonstrate the efficacy of this strategy in mouse USH1F models, effectively restoring hearing and balance in these mice. Importantly, our approach also proves successful in expressing PCDH15 protein in clinically relevant retinal models, including human retinal organoids and nonhuman primate retina, showing efficient targeting of photoreceptors and proper protein expression in the calyceal processes. This research represents a major step toward advancing gene therapy for USH1F and the multiple challenges of hearing, balance, and vision impairment.

## Abstract 2

In this work we developed dual AAVs gene therapy for Usher syndrome type 1F, a genetic disorder causing hearing loss, balance issues, and vision impairment.

## Graphical abstract

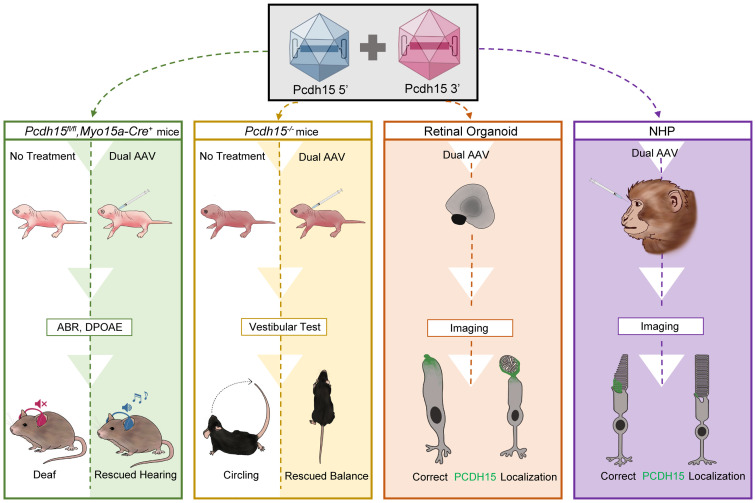

## Introduction

Usher syndrome type 1F (USH1F) is a debilitating genetic condition in which patients are born without the senses of hearing or balance and further experience gradual deterioration of vision (1–7). The disparate symptoms of USH1F result from dysfunction in a single gene that encodes the extracellular adhesion protein protocadherin-15 (PCDH15) (5, 8). In the cochlea, PCDH15 is essential for mediating hair cell mechanotransduction, accounting for the dramatic loss of hearing and balance associated with mutations (9–11). Vision impairment in USH1F is due to retinitis pigmentosa, a progressive peripheral-to-central degeneration of the rod and cone photoreceptors of the retina (12–14). Most USH1F patients experience loss of night vision in their teens, which eventually progresses to tunnel vision and complete blindness (15). The loss of vision is particularly devastating because patients rely on vision to compensate for the impairment of hearing and balance. Furthermore, the delayed manifestation of these symptoms presents a therapeutic window during which intervention may be effective, prior to photoreceptor death.

Over the last 20 years, we and others have explored the role of PCDH15 in the cochlea, elucidating its importance for mechanotransduction and hair cell development (5, 6, 9, 10, 16–22). However, the role of PCDH15 in the retina has remained less clear. While mouse models of USH1F faithfully recapitulate auditory and vestibular defects, they exhibit only subtle changes in vision function and no obvious change in retinal morphology (12). Studies in amphibians and nonhuman primates have indicated that PCDH15 is a component of the calyceal processes (23, 24) that surround the bases of photoreceptor outer segments and maintain their integrity. Importantly, calyceal processes are absent from mouse photoreceptors, which may explain the limited visual phenotype of mouse models of USH1F (23).

Clinically, USH1F is an attractive candidate for intervention by gene therapy: subretinal adeno-associated virus (AAV) therapy was approved for clinical use (25), and many other retinal gene therapies are in development (26). However, the large coding sequence for PCDH15 constrains an AAV-based gene delivery. Recently, we effectively circumvented this constraint in the cochlea using two gene delivery mechanisms: a mini-gene approach (27) and a base-editing approach (28). For those studies, we generated two mouse models of USH1F cochlear pathology, a *Pcdh15*-floxed mouse with recombination induced by *Myo15a-Cre* and a humanized mouse line bearing the common pathogenic R245X truncating mutation flanked by the human coding sequence. In this study, we use these models as well as human retina organoids and a primate retina to test a third circumvention of the length constraint: dual-AAV gene delivery.

Dual-AAV gene delivery relies on cotransduction of a single cell with 2 different viruses, each carrying half of the genetic cargo, with subsequent intracellular recombination. Here, we use a hybrid dual-AAV strategy that incorporates both trans-splicing and overlapping elements to maximize the proper concatemerization of the viral DNA (29–33). In recent years, this strategy has been used to deliver larger transgenes to the cochlea, such as *OTOF* (31, 34–36) and *MYO7A* (37), and to the retina, such as *ABCA4* (38) and *MYO7A* (39). Using our mouse models, we demonstrate remarkable efficacy in correcting hearing and balance defects. For potential retinal therapy, we tested the dual-AAV therapy in 2 clinically relevant models: human retinal organoids and retina of the green monkey, an Old World primate. In both human and monkey, we demonstrate safe and efficient targeting of photoreceptors, as well as proper protein expression and localization to the calyceal processes. Our study offers promise for correcting USH1F-associated defects in hearing, balance, and vision.

## Results

### Generation of dual AAV vectors and verification in HEK293 cells.

**Figure 1:**
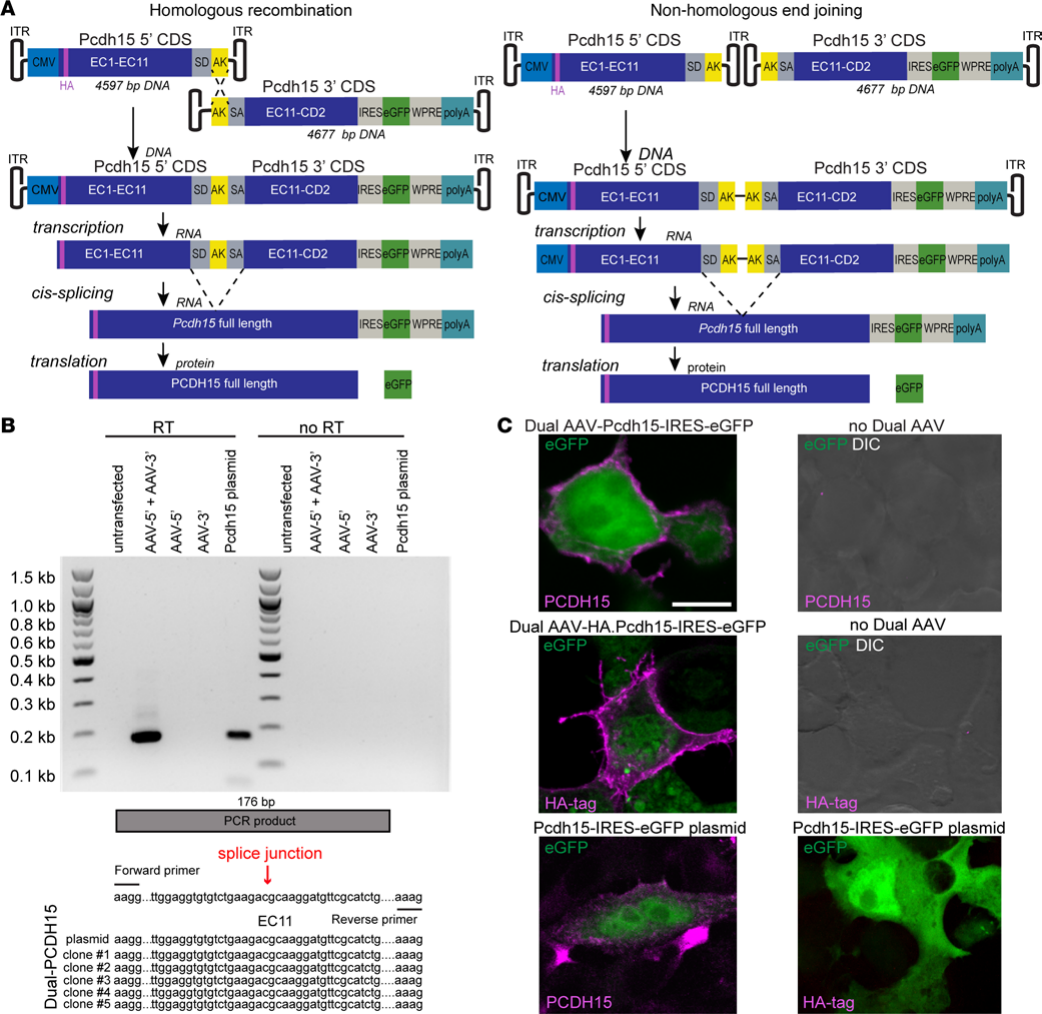
Splicing and protein production in vitro. (**A**) Recombination and splicing of dual vectors to produce full-length protein. The reassembly is mediated by non-homologous end joining of the inverted terminal repeats (ITRs) and/or homologous recombination of the highly recombinogenic (AK) sequence. After transcription, splicing can occur from the splice donor site (SD) in vector 1 to the splice acceptor site (SA) in vector 2, creating a full-length *Pcdh15* mRNA. (**B**) mRNA was obtained from HEK cells and reverse-transcribed (*n* = 5). A cDNA library was Sanger-sequenced around the splice junction, confirming proper recombination and splicing. (**C**) Dual AAVs (encoding PCDH15 with or without an N-terminal HA tag) were added to HEK293 cell cultures (*n* = 3 per group). Immunostaining was performed with anti-PCDH15 or anti-HA antibodies (magenta). Representative confocal images demonstrate normal trafficking of PCDH15, either with or without the N-terminal HA tag, to the cell membrane after dual-AAV delivery. Labeling was similar to that with transfection of a single plasmid encoding full-length PCDH15. No specific signal was detected in the control samples (*n* = 3 per group). Scale bar: 10 μm. CDS, coding sequence; CMV, cytomegalovirus; DIC, differential interference contrast; IRES, internal ribosome entry site; polyA, polyadenylation signal; WPRE, woodchuck hepatitis virus post-transcriptional regulatory element.

For testing gene replacement therapy in the USH1F mouse inner ear, we used the mouse *Pcdh15-CD2* isoform (NM_001142742.1), previously confirmed to be necessary for PCDH15 function in hair cells in mice (17, 40). *Pcdh15-CD2* has an approximately 5.3-kb coding sequence, too large to fit in a single AAV capsid. We engineered dual-AAV hybrid vectors so that each vector encoded about half of the full-length protein. Vector 1 (AAV-Pcdh15 5′) included a CMV promoter, the N-terminal half of the *Pcdh15* coding sequence, and a splice donor (SD) site followed by a highly recombinogenic sequence from F1 phage (AK) (32). Vector 2 (AAV-Pcdh15 3′) included the AK sequence, a splice acceptor (SA), the C-terminal half of the *Pcdh15* coding sequence, and other regulatory elements (Figure 1A). Once AAV vectors are in the same cell, the reassembly is mediated by homologous recombination of the AK sequence and/or non-homologous end joining of the inverted terminal repeats. SD and SA sites facilitate the excision of the AK sequence, producing full-length *Pcdh15* mRNA and PCDH15 protein (Figure 1A) (30, 41). This method has worked well for the dual-AAV expression of otoferlin in the cochlea (31, 34–36, 42). Each recombinant vector was packaged in the AAV9-PHP.B capsid, which efficiently transduces both inner hair cells (IHCs) and outer hair cells (OHCs) of the cochlea (43–45). We also made a second N-terminal vector that includes a hemagglutinin (HA) epitope tag at the N-terminus of PCDH15 (AAV-HA.Pcdh15 5′). The N-terminus is a short helix extending away from the first EC domain and away from the bond with cadherin-23 (CDH23), so the HA tag itself does not interfere with normal binding (27).

We first assessed splicing and protein production in vitro. An HEK293 cell line was transduced with either AAV-Pcdh15 5′ + AAV-Pcdh15 3′ or AAV-HA.Pcdh15 5′ + AAV-Pcdh15 3′. Cells were collected for mRNA or fixed for immunofluorescence. mRNA harvested from HEK293 cells was reverse-transcribed, and a cDNA library was Sanger-sequenced around the splice junction, confirming proper recombination and splicing (Figure 1B). We labeled fixed cells with antibodies against PCDH15 or against the HA tag. If, and only if, cells were transduced with both AAVs, we observed strong labeling of the plasma membranes with either anti-PCDH15 or anti-HA (Figure 1C). Labeling was the same as with transfection of a single plasmid encoding full-length PCDH15, indicating that recombination and splicing were efficient.

### Dual-AAV–mediated Pcdh15 coding sequence delivery rescues auditory and vestibular function in the USH1F mouse model and shows no toxicity for hearing.

To test efficiency of dual-AAV delivery in rescue of hearing, we used *Pcdh15^fl/fl^* conditional knockout mice, in which exon 31 — encoding PCDH15’s transmembrane domain and part of the MAD12 domain — was flanked by *loxP* sites (27). Exon 31 deletion by recombination occurred in hair cells when mice also carried a Cre recombinase driven by the late-onset, hair cell–specific *Myo15a* promoter, for which expression starts at P0 (46). As previously shown, *Pcdh15^fl/fl^*, *Myo15a-Cre^+/–^* mice exhibited no major abnormalities in cochlear hair bundle morphology at P6. Most middle-row stereocilia had elongated tips, suggesting actin core remodeling, but hair cell labeling by the fluorescent dye FM1-43, which passes through open mechanotransduction channels, indicated nearly normal mechanotransduction, similar to that in control mice (27). However, at 5 weeks of age, mice lacked any identifiable auditory brainstem response (ABR) to loud sound stimulation across the full frequency spectrum tested, indicating profound hearing loss (27). Distortion-product otoacoustic emission (DPOAE) responses at 5 weeks of age, diagnostic of OHC function, were largely absent at all sound intensities and frequencies tested. Scanning electron microscopy and fluorescent phalloidin staining of actin revealed severely disrupted bundles: middle- and short-row stereocilia of IHCs and OHCs in the *Pcdh15^fl/fl^*, *Myo15a-Cre^+/–^* mice were shortened or totally missing. Hair cells also showed no detectable FM1-43 loading in adults, confirming no functional mechanotransduction (27).

**Figure 2:**
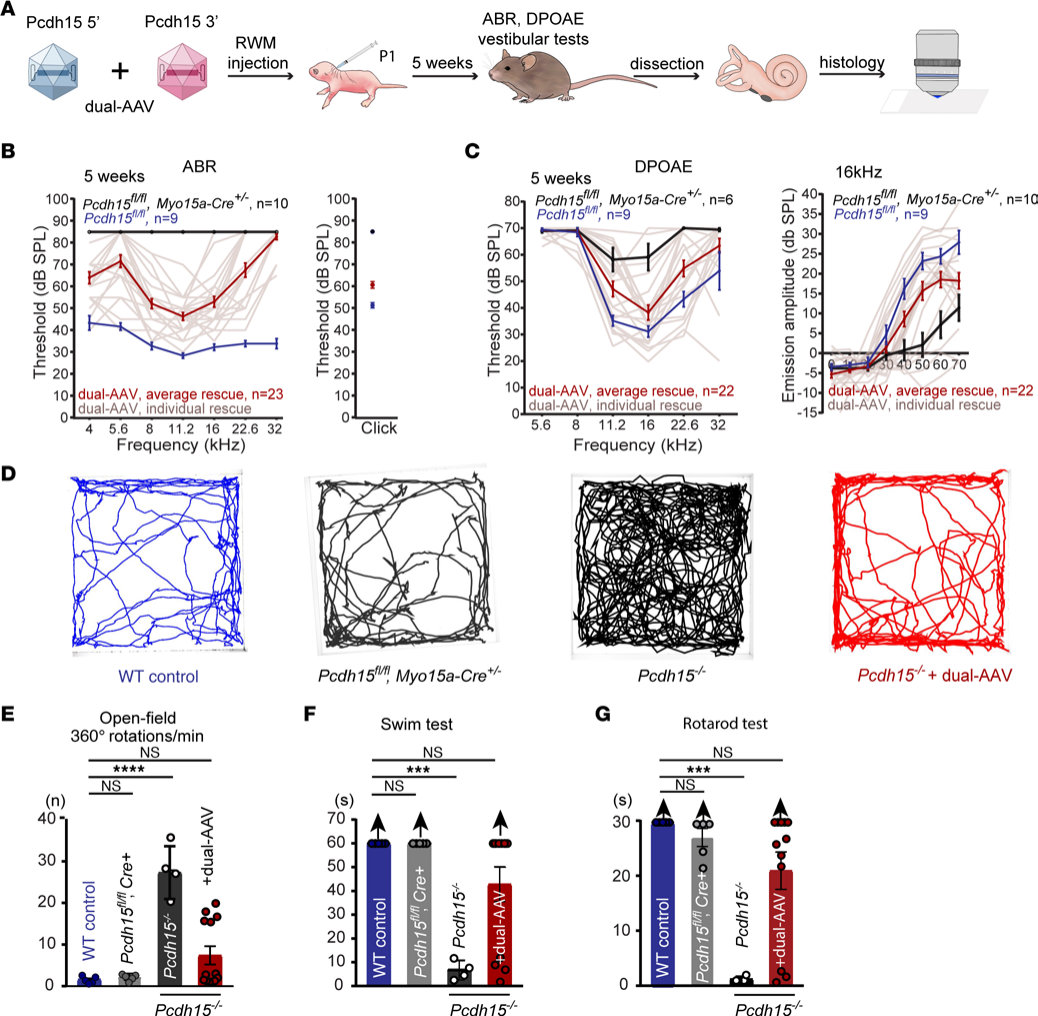
Dual-AAV delivery of PCDH15 successfully preserves hearing in *Myo15a-Cre* mouse model and alleviates vestibular deficits in constitutive knockout model of USH1F. (**A**) Dual AAV vectors encoding PCDH15-CD2 protein were injected via the RWM into either conditional or constitutive knockout mice at P1. At 5 weeks, treated animals were assayed for hearing and vestibular function and processed for histology. (**B**) ABR click and tone thresholds for untreated *Pcdh15^fl/fl^* Cre control mice (*n* = 9), untreated *Pcdh15^fl/fl^*, *Myo15a-Cre^+/−^* mice (*n* = 10), and treated *Pcdh15^fl/fl^*, *Myo15a-Cre^+/−^* mice (*n* = 23). (**C**) Average DPOAE thresholds and amplitudes at 16 kHz for 5-week-old hearing control mice (*n* = 9), for untreated *Pcdh15^fl/fl^*, *Myo15a-Cre^+/−^* mice (*n* = 6), and treated *Pcdh15^fl/fl^*, *Myo15a-Cre^+/−^* mice (*n* = 22). (**D**) Representative open field path outlines of wild-type mice, untreated *Pcdh15^fl/fl^*, *Myo15a-Cre^+/−^* mice, *Pcdh15^–/–^* knockout mice, and treated *Pcdh15^–/–^* knockouts. (**E**) Summary of total rotations in wild type (*n* = 5), untreated *Pcdh15^fl/fl^*, *Myo15a-Cre^+/−^* mice (*n* = 5), *Pcdh15^–/–^* mice (*n* = 4), and treated *Pcdh15^–/–^* mice (*n* = 13). Untreated *Pcdh15^–/–^* mice exhibited severe circling behavior, while vestibular function was fully preserved at normal levels in dual AAV-treated *Pcdh15^–/–^* mice. (**F**) Wild-type (*n* = 5) and *Pcdh15^fl/fl^*, *Myo15a-Cre^+/−^* (*n* = 5) mice swam well. *Pcdh15^–/–^* mice (*n* = 4) were unable to swim normally. **G**) *Pcdh15^–/–^* mice showed much shorter latency to fall on the rotarod test (*n* = 4), while treated *Pcdh15^–/–^* mice performed at wild-type levels (*n* = 12). One-way ANOVA followed by Dunnett’s test was used to assess statistical significance. Data are presented as mean ± SEM. ***P < 0.001, ****P < 0.0001; n.s., not significant (*P* > 0.05).

AAV vectors were injected into cochleae of *Pcdh15^fl/fl^*, *Myo15a-Cre^+/–^* conditional knockout mice at P1 via the round window membrane (RWM), with a dose of 5.0 × 10^10^ vector genome copies (VGC) of each vector (1.0 × 10^11^ VGC total). At 5 weeks, the treated animals were tested physiologically to determine the extent of hearing rescue, and cochlear tissue was processed for histological examination (Figure 2A).

We first assessed hearing in treated mice by recording ABRs to broadband clicks and tone bursts (Figure 2B). We found that *Pcdh15^fl/fl^*, *Myo15a-Cre^+/–^* mice injected at P1 with dual AAVs encoding either PCDH15 or HA.PCDH15 showed robust rescue of hearing (Figure 2B and Supplemental Figure 1A). The thresholds in rescued animals treated with PCDH15, either with or without the HA tag, were the same (Supplemental Figure 1A), confirming that HA-tagged PCDH15 is fully functional. In some mice, hearing was near that of untreated *Pcdh15^fl/fl^* control mice lacking Cre. Untreated *Pcdh15^fl/fl^*, *Myo15a-Cre^+/–^* mice were deaf at 5 weeks as tested by ABR, with thresholds above 80 dB, the highest level tested. *Pcdh15^fl/fl^* normal-hearing control mice injected with dual AAVs had normal thresholds indicating no sign of toxicity for hearing (Supplemental Figure 1B).

DPOAE measurements also demonstrated strong rescue in dual-AAV–treated mice (Figure 2C and Supplemental Figure 1A), with thresholds near those of hearing control *Pcdh15^fl/fl^* Cre^–^ mice. Similarly, DPOAE amplitudes measured at a representative midrange frequency (16 kHz) showed rescue in treated *Pcdh15^fl/fl^*, *Myo15a-Cre^+/–^* conditional knockout mice to near the hearing control level. We observed that *Pcdh15^fl/fl^* control mice injected with the dual AAVs exhibited normal DPOAE thresholds, further indicating absence of toxicity (Supplemental Figure 1B). These experiments use a late-deletion mouse model, which allows normal hair cell development before *Pcdh15* gene deletion, but the model may not represent the typical phenotype of USH1F patients, who lack PCDH15 at all ages. The experiments nevertheless show that dual-AAV delivery produces a functional PCDH15 and preserves hearing.

Surprisingly, *Pcdh15^fl/fl^*, *Myo15a-Cre^+/–^* mice did not show a behavioral vestibular deficit, assessed by swimming, open field locomotion, and rotarod tests (Figure 2, D–G). We previously speculated that the presence of functional PCDH15 in the first postnatal week allowed visual and proprioceptive input to be linked with vestibular sensation, so that the mice could adequately perform locomotory tasks even after deletion of *Pcdh15* using vision and proprioception (28). To test efficiency of dual-AAV delivery of *Pcdh15* in rescue of vestibular function in a more severe model, we used a constitutive knockout mouse model bearing the R245X truncation mutation (*Pcdh15^R245X/R245X^*, here referred to as *Pcdh15^–/–^*) (28). These mice exhibited a severe vestibular phenotype including intensive head bobbing, circling behavior, hyperactivity, difficulty swimming, and inability to remain on a turning rotarod (Figure 2, D–G) (28). AAV vectors were administered via the RWM into *Pcdh15^–/–^* mice at P1, with a dose of 1.0 × 10^11^ VGC. In mice 5 weeks of age, we assessed vestibular function rescue with swimming, open field locomotion, and rotarod tests. In all 3 tests, the dual-AAV delivery of full-length PCDH15-CD2 preserved function at wild-type levels (Figure 2, D–G). However, no ABR was detected in these mice, even at the highest level tested, 80 dB sound pressure level (SPL).

### Dual-AAV delivery of Pcdh15-CD2 preserved stereocilia bundle morphology, tip links, and mechanotransduction in the Pcdh15^fl/fl^, Myo15a-Cre^+/–^ conditional knockout mouse.

**Figure 3:**
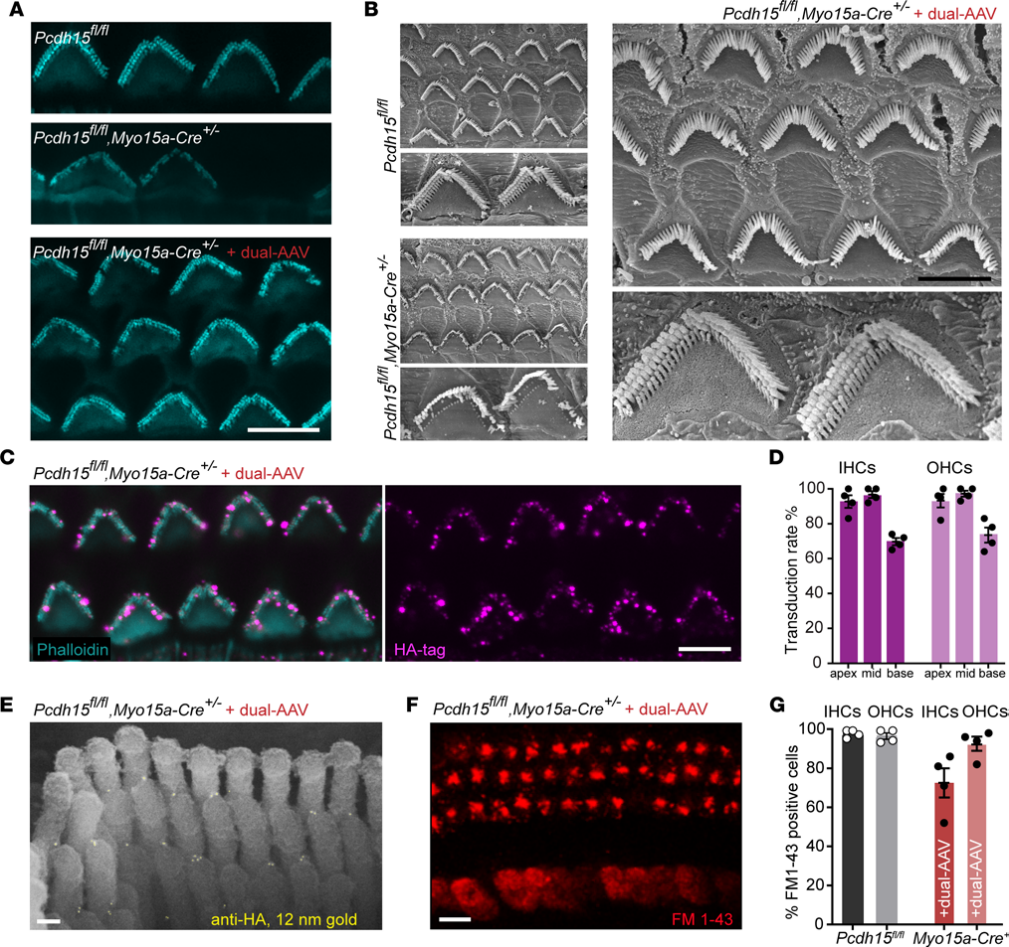
Delivery of PCDH15 with dual AAVs preserves the morphology of stereocilia bundles, mechanotransduction, and tip links in the *Pcdh15^fl/fl^*, *Myo15a-Cre^+/−^* conditional knockout mouse model. (**A**) Representative confocal microscopy images captured from the middle turn of 5-week-old cochleae, showing OHCs stained with phalloidin. The panels illustrate OHCs from *Pcdh15^fl/fl^* hearing control mice (*n* = 3), uninjected *Pcdh15^fl/fl^*, *Myo15a-Cre^+/−^* conditional knockout mice (*n* = 3), and knockout mice that received dual-AAV injections (*n* = 5). (**B**) Scanning electron micrographs of hair bundles of a hearing control mouse and untreated conditional knockouts. In the knockouts, the bundles were severely disrupted. However, in the OHCs of a conditional knockout treated with dual AAVs encoding PCDH15, normal bundle morphology was preserved (*n* = 5). (**C**) Representative confocal microscopy images at 5 weeks from the middle turn of the cochlea show anti-HA staining at the tips of stereocilia in knockout cochleae treated with dual AAVs encoding HA.PCDH15 at P1 (*n* = 4). (**D**) Transduction efficiency in IHCs and OHCs at 5 weeks in treated *Pcdh15^fl/fl^*, *Myo15a-Cre^+/−^* conditional knockout mice (*n* = 4). Data are presented as mean ± SEM. (**E**) Immunogold scanning electron microscopy localization of HA-tagged PCDH15 in OHC stereocilia of treated knockout mice. Multiple 12 nm gold beads (light yellow) were detected in a scanning electron microscopy image, confirming that HA-tagged PCDH15 goes to the tips of stereocilia, except the tallest (*n* = 3). (**F**) Rescue of FM1-43 uptake in a treated cochlea. (**G**) Average percentage of IHCs and OHCs loaded with FM1-43 in conditional knockout mice injected with dual AAVs (*n* = 4) and in control mice (*n* = 4). Data are presented as mean values ± SEM. Scale bars: 5 μm (**A**–**C** and **F**), 100 nm (**E**).

Next, we assessed stereocilia bundle morphology and hair cell mechanotransduction using dual-AAV delivery, employing both a fluorescent phalloidin actin label and scanning electron microscopy. Actin labeling of hair bundles revealed that at 5 weeks, untreated conditional knockout mice exhibited disorganized bundles or had lost their hair bundles entirely (Figure 3, A and B). This disorganization included the loss of short- and middle-row stereocilia, with only some of the tall-row stereocilia remaining. In contrast, dual-AAV delivery of *Pcdh15-CD2* led to robust preservation of hair cell morphology, with bundles at 5 weeks closely resembling those in *Pcdh15^fl/fl^* Cre^–^ control mice (Figure 3, A and B).

The successful preservation of both hearing (Figure 2, B and C) and bundle morphology further indicates that PCDH15-CD2 protein is effectively expressed and accurately targeted within the hair cells. To assess the proper trafficking and localization of exogenous PCDH15 at the tips of cochlear stereocilia, we used the AAV-HA.Pcdh15 5′ + AAV-Pcdh15 3′ vector pair, which incorporates an HA tag at the N-terminus of PCDH15. Immunofluorescence imaging confirmed that conditional knockout mice injected at P1 with AAV-HA.Pcdh15 5′ + AAV-Pcdh15 3′ exhibited robust immunoreactivity to the HA tag. Importantly, this labeling was located at the tips of stereocilia (Figure 3C) in treated cochleae but not in the controls, confirming the antibody specificity (Supplemental Figure 2A). Hair cell expression of HA.PCDH15 varied from apex to base, with 93%–98% of apical and middle IHCs and OHCs transduced and 70%–74% of basal hair cells transduced (Figure 3D).

With scanning electron microscopy and a secondary antibody conjugated to 12 nm gold beads, we observed the location of HA.PCDH15 within hair bundles. Gold beads were specifically localized on the tips of short- and middle-row stereocilia, precisely at the position of the tip links (Figure 3E). This confirms the accurate trafficking and localization of HA.PCDH15 at age 5 weeks. No gold beads were detected in the control samples (Supplemental Figure 2B).

In order to evaluate the preservation of mechanotransduction in hair cells with dual-AAV delivery, we evaluated hair cell loading of FM1-43. We dissected apical and mid-apical regions of the cochleae at 5 weeks, briefly applied FM1-43 dye directly onto the exposed epithelium, and subsequently applied a wash solution of 4-sulphonate calix[4]arene, sodium salt (SCAS), which acts as a chelator for FM1-43 (Figure 3F). We quantified the proportion of FM1-43–positive cells in the cochlea. FM1-43 labeling revealed a preservation of mechanotransduction in approximately 93% of OHCs and 73% of IHCs (Figure 3G), in comparison with untreated animals, in which no detectable FM1-43 signal was observed (Supplemental Figure 2C).

### PCDH15 gene structure and disease-associated variants.

*PCDH15* is expressed as one of 3 primary transcripts, namely the *CD1*, *CD2*, and *CD3* isoforms. These isoforms differ primarily in the 3′ end of the gene. The CD2 splice form is necessary for PCDH15 function in hair cells (17, 40), though it remains uncertain which splice form is predominant in the retina. In order to investigate this, we analyzed *PCDH15* exon structure and human disease-associated variants.

**Figure 4:**
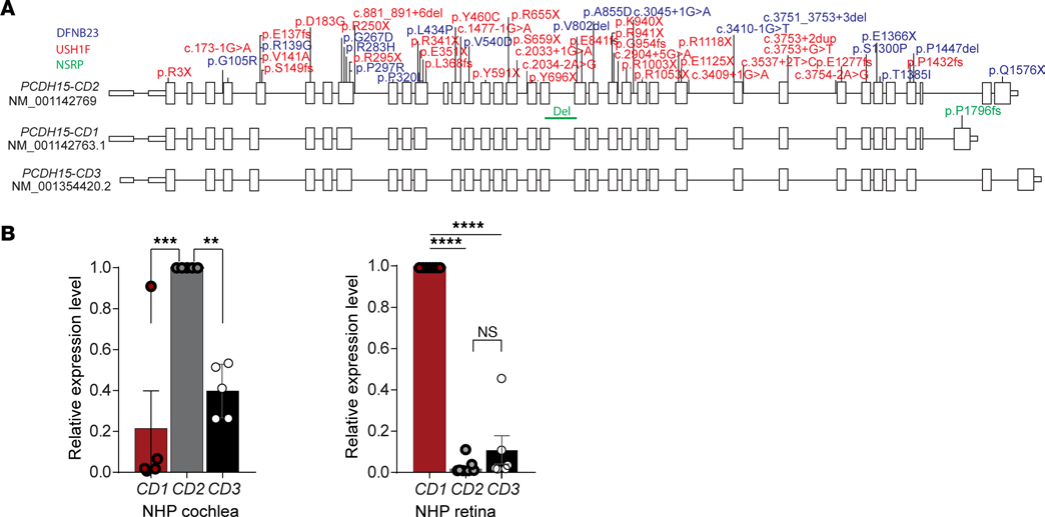
Human *PCDH15* exon structure and disease-associated variants. (**A**) Gene schematic of *PCDH15-CD2* and reported disease-causing variants. Three primary transcripts of *PCDH15* (*CD1*, *CD2*, and *CD3*) translate 3 isoforms, which differ primarily in the 3′ end of the gene. Disease variants were gathered from the Deafness Variation Database, accessed August 2021. Disease variants are plotted based on their location on transcript NM_001142769 and color-coded based on associated disease phenotype: blue, non-syndromic hearing loss (DFNB23); red, Usher syndrome type 1F (USH1F); or green, non-syndromic retinitis pigmentosa (NSRP). Note that the variant p.P1796fs impacts the protein-coding sequence of only the *CD1* isoform and has been reported to cause NSRP, suggesting that the CD1 splice form is required for retinal function but not hearing. Similarly, p.Q1576X in *CD2* affects hearing but not vision. (**B**) Quantitative PCR evaluation of *PCDH15-CD1*, *PCDH15-CD2*, and *PCDH15-CD3* expression in primate cochlea (*n* = 5) and retina (*n* = 6). One-way ANOVA followed by post hoc Tukey’s test was used to assess statistical significance. Data are presented as mean ± SEM. **P < 0.01, ***P < 0.001, ****P < 0.0001; n.s., not significant (*P* > 0.05).

Disease-associated variants were systematically collected from the Deafness Variation Database (47), accessed in August 2021. These variants were then mapped to their respective positions on the human NM_001142769 transcript and color-coded based on the associated disease phenotype: variants associated with non-syndromic hearing loss (DFNB23), variants linked to USH1F with both deafness and blindness, and variants connected with non-syndromic retinitis pigmentosa (NSRP). It is noteworthy that a specific variant, p.P1796fs, impacts the protein-coding sequence exclusively within the *CD1* isoform. This variant has been documented as a causative factor for NSRP (48), suggesting a fundamental role of the CD1 splice form in retinal function (Figure 4A).

We performed quantitative reverse transcription PCR on an inner ear and eye of a cynomolgus monkey (*Macaca fascicularis*) to reveal tissue-specific *PCDH15* isoform expression. In the nonhuman primate (NHP) inner ear, the predominant splice form of the C-terminal region was identified as *CD2*, with some detectable expression of *CD3* (Figure 4B). Within the NHP retina, expression of *CD1* was higher than that of *CD2*; here too there was some *CD3* expression. These findings highlight the tissue-specific variation in *PCDH15* isoform expression, with *CD2* being prominent in the inner ear and *CD1* in the NHP retina.

### Evaluation of dual AAV in human retina organoids.

**Figure 5:**
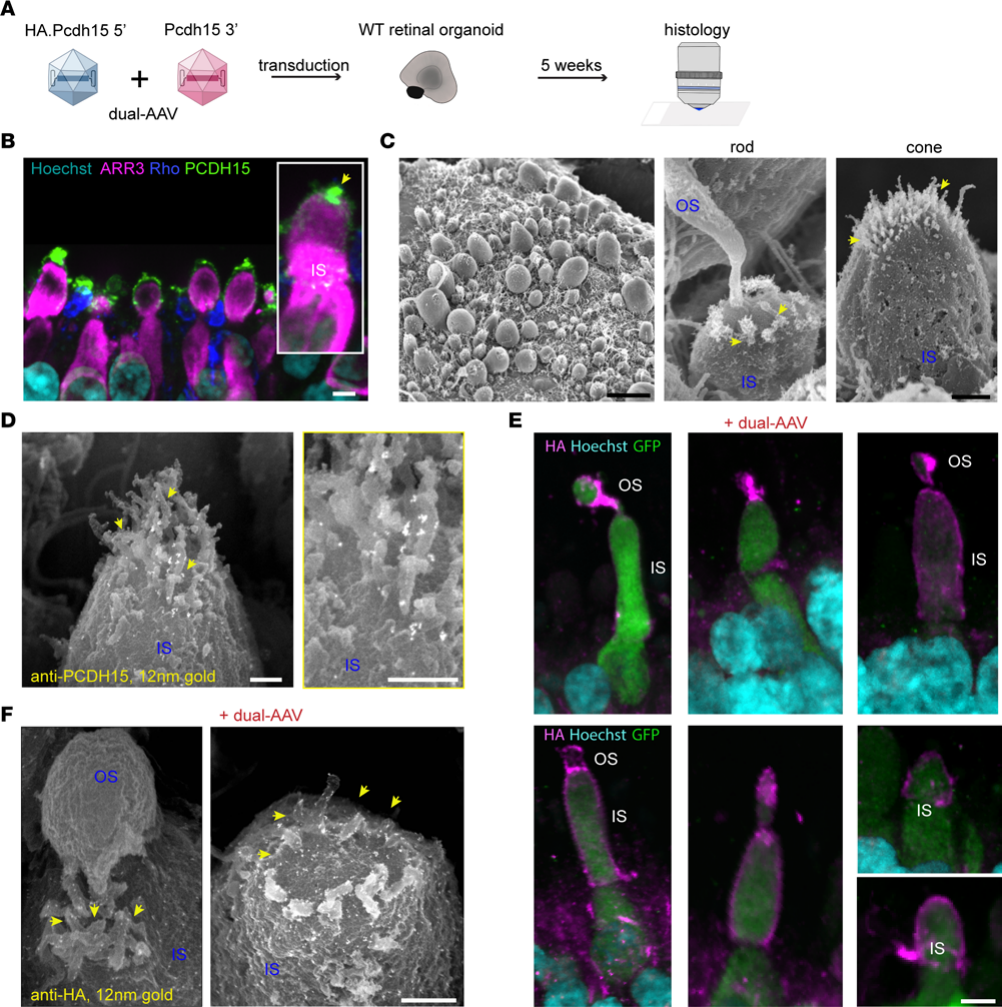
Expression of PCDH15 in human retinal organoids by dual AAV vectors. (**A**) Organoids were transduced with dual AAV vectors, and 5 weeks later expression was analyzed by antibodies against the HA tag. (**B**) Representative confocal microscopy images captured from cryosections of non-transduced human retina organoids. Anti-PCDH15 labeling (green) was detected in the photoreceptors, especially at the distal ends of outer segments (OSs) (yellow arrows) (*n* = 3 organoids). Hoechst dye labeled nuclei, anti–cone arrestin (ARR3) labeled cones, and anti-rhodopsin (Rho) labeled rod OSs. (**C**) Scanning electron microscopy of human retinal organoids. The finger-like calyceal processes (yellow arrows) protrude from the apical region of the inner segments (ISs) of both rod (middle) and cone (right) photoreceptors (*n* = 3 organoids). (**D**) Immunogold scanning electron microscopy labeling of non-transduced human retinal organoids immunostained with anti-PCDH15 primary antibody and 12 nm gold-conjugated secondary antibody. Endogenous PCDH15 was located at the surfaces of ISs and of nascent calyceal processes of photoreceptors (*n* = 3 organoids). (**E**) Anti-HA fluorescent labeling of vector-delivered HA.PCDH15. Because the CMV promoter is only present on the 5′ vector, HA.PCDH15 and GFP were only expressed when both the 5′ and 3′ vectors were added to the organoids and were internalized within a cell. Anti-HA.PCDH15 is shown as magenta and GFP as green. In these images, GFP diffusely labeled doubly transduced cells. HA.PCDH15 is seen at the junction of ISs and OSs of cone photoreceptors (*n* = 3 organoids). (**F**) Immunogold scanning electron microscopy confirmed localization of HA-tagged hsPCDH15 to calyceal processes and ISs of human retinal organoids transduced with dual-AAV (*n* = 3). Multiple 12 nm gold beads were detected. Scale bars: 5 μm (**B** and **E**), 10 μm (**C**, left), 1 μm (**C**, right), 0.5 μm (**D** and **F**).

Retinal degeneration in USH1F patients occurs over decades, providing a window of opportunity for the treatment of retinal degeneration. Gene therapy could target degenerating photoreceptors in the retina to halt visual field loss, but it requires testing of a therapeutic construct in a model that is relevant to the human retina. We derived retinal organoids from human induced pluripotent stem cells and tested gene delivery using the dual-AAV vector strategy (Figure 5A). First, we examined photoreceptor ultrastructure within retinal organoids and the localization of PCDH15 within photoreceptors. Immunofluorescence imaging of cryosections labeled with anti-PCDH15 antibodies revealed distinct staining in the photoreceptors, especially at the distal ends of the inner segments (ISs) (Figure 5B). Additionally, scanning electron microscopy analysis revealed that the majority of photoreceptors exhibited well-developed ISs, while a subset displayed outer segments (OSs) and connecting cilia. Notably, we observed the emergence of nascent calyceal processes at the apical ends of the ISs (Figure 5C). Further investigation using immunogold scanning electron microscopy of human retinal organoids that were subjected to immunostaining with anti-PCDH15 primary antibodies and subsequent labeling with 12 nm gold-conjugated secondary antibodies demonstrated the trafficking of PCDH15 to the surfaces of the ISs and the nascent calyceal processes of the photoreceptors (Figure 5D).

Next, we packaged CMV-GFP in an AAV9-PHP.B capsid. Human retinal organoids were incubated with 8.5 × 10^11^ VGC and evaluated 5 weeks later for GFP expression (Figure 5A). We found that AAV9-PHP.B robustly transduced photoreceptors of retinal organoids (Supplemental Figure 3A). For gene expression studies in retina, we used the human *PCDH15-CD1* isoform (NM_001142763.1) tagged with HA. We engineered dual-AAV hybrid vectors so that each vector encoded part of the full-length protein. As for mouse vectors, vector 1 (AAV-HA.Pcdh15 5′) included a CMV promoter, the N-terminal half of the *PCDH15* coding sequence with HA tag, and a splice donor site followed by the AK recombinogenic sequence. Vector 2 (AAV-Pcdh15 3′) included the AK sequence, a splice acceptor, the C-terminal half of the *PCDH15* coding sequence, and the GFP coding sequence following an internal ribosomal entry site (IRES). We transduced retinal organoids derived from human induced pluripotent stem cells using these dual AAVs, separately or together. After 5 weeks, we assessed the expression of GFP and HA-tagged PCDH15 using immunofluorescence and immunogold scanning electron microscopy imaging. Because the organoids presumably expressed the endogenous human PCDH15, we detected the AAV-delivered PCDH15 with antibodies against HA rather than against PCDH15 itself (Figure 5, E and F).

Notably, we did not detect the presence of the HA tag when organoids were transduced with either AAV-HA.Pcdh15 5′ or AAV-Pcdh15 3′ alone (Supplemental Figure 3, B and C). However, anti-HA antibody labeling revealed expression of HA.PCDH15 in organoids transduced with the dual AAVs, and further showed that the HA tag was specifically localized on the surfaces of the ISs and at the junction between the ISs and OSs, precisely where the development of calyceal processes occurs (Figure 5, E and F). This observation confirmed that the dual-AAV approach, initially developed for the inner ear, successfully transduced and expressed full-length PCDH15 in photoreceptors, with specific localization to the calyceal processes and ISs of photoreceptors (Figure 5, E and F).

### Evaluation of dual-AAV delivery in NHP retina.

**Figure 6:**
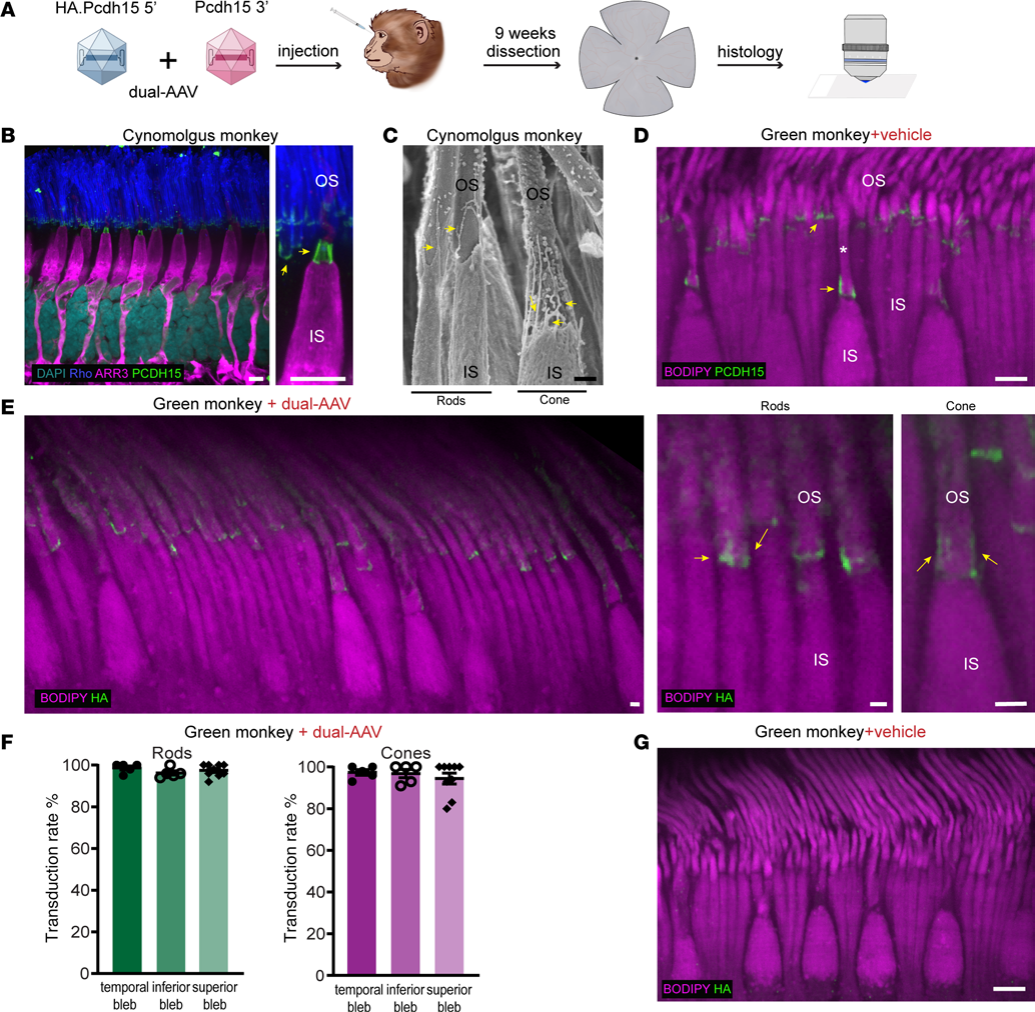
Localization of endogenous and dual-AAV–delivered PCDH15 in NHP retina. (**A**) Dual AAV vectors were injected subretinally into the eye of a green monkey (*C*. *sabaeus*). At 9 weeks, the treated retina was assayed for expression and localization by histology. (**B**) Immunofluorescence labeling of endogenous PCDH15 (green) in the retina of a cynomolgus monkey (*M*. *fascicularis*). An antibody against ARR3 (magenta) marks cone photoreceptors, and an antibody against Rho (blue) marks OSs of rods. PCDH15 (green) is located at the junction between ISs and OSs and along the calyceal processes (arrows) in both cones and rods (*n* = 4 punches). (**C**) Scanning electron micrograph of the inner/outer segment junction in a cone photoreceptor (right) and rod photoreceptors (left) in a cynomolgus monkey (*n* = 4 punches). The calyceal processes (arrows) protrude from the apical ISs of photoreceptors to surround the OSs. (**D**) Immunofluorescence labeling of endogenous PCDH15 (green) in the vehicle-injected control retina of a green monkey (*n* = 3 punches). PCDH15 (green) is located at the junction between ISs and OSs and along the calyceal processes (arrows) in both cones and rods. White asterisk indicates the OS of a cone photoreceptor. BODIPY labels all membranes of photoreceptors. (**E**) Representative confocal microscopy images of cryosections from the bleb area created by the dual-AAV injection. An anti-HA signal was detected in the photoreceptors injected with dual AAVs, seen at low magnification (left) and higher magnification (right two panels). (**F**) Transduction efficiency in rod and cone photoreceptors injected with dual AAVs. Data are presented as mean ± SEM. (**G**) The HA label was not seen in the eye injected with the vehicle (*n* = 3 blebs). Scale bars: 5 μm (**B**, **D**, and **G**), 1 μm (**C** and **E**).

Finally, we assessed the efficiency of dual-AAV delivery in NHPs, which represent the most pertinent animal model for transgene delivery to the human eye. In the green monkey (*Chlorocebus sabaeus*), we administered dual-AAV9-PHP.B vectors by subretinal injection, a well-established and effective route for delivering therapeutic agents to the retina. In the eyes, the subretinal injection caused a focal area of bullous retinal detachment, commonly referred to as a bleb. The formation of the bleb is an essential part of the gene therapy delivery process, indicating successful injection and distribution of the therapeutic vector to the proper retinal layer. Specifically, in one eye AAV injection produced 3 blebs in the superior, inferior, and temporal regions of the retina, each containing dual AAV at a dosage of 2.5 × 10^12^ VCG per bleb (totaling 7.5 × 10^12^ VCG per eye), with each bleb containing 50 μL of virus. The second eye served as a control and received an injection of a formulation buffer consisting of PBS/0.001% F-68, with the same distribution of 3 blebs in the superior, inferior, and temporal regions of the retina, each containing 50 μL. Before the injection procedure, the monkey underwent a comprehensive baseline evaluation. The general assessment included the determination of AAV9 neutralizing antibody seronegativity, an evaluation of general well-being, and a thorough examination of ocular health. To verify the successful formation of subretinal blebs, we used optical coherence tomography immediately after the surgical procedure. The subretinal bleb flattened over the following days, with retinal reattachment occurring in all cases. In general, the procedure, performed under anesthesia, was well tolerated by the animal, which was closely monitored for a duration of 9 weeks after injection. Subsequently, we conducted an analysis of retinal tissues using histological methods (Figure 6A).

Initially, we conducted a detailed examination of the photoreceptors by immunolabeling the retina of a cynomolgus monkey — a related species — with antibodies specific to cone arrestin and rhodopsin (Figure 6B). The cone arrestin counterstain provided insights into the comprehensive cone anatomy, revealing well-preserved structures that closely matched those observed in scanning electron microscopy images (Figure 6C). PCDH15 staining formed barrel-like structures surrounding the bases of the OSs (Figure 6B), which correspond to the calyceal processes seen in scanning electron microscopy. In contrast, rhodopsin labeled the OSs of rod cells, where PCDH15 was observed as a thin layer at the bases of the OSs, with a few extensions along the OSs (Figure 6B). This pattern correlates with the fewer number of calyceal processes in rods compared with cones (Figure 6C).

Next, we determined the normal localization of PCDH15 in photoreceptors of the control eye of a green monkey injected with PBS, using an anti-PCDH15 antibody and the fluorescent dye BODIPY, which labels lipid membranes including those in cones and rods. Our findings confirmed that PCDH15 localizes, as previously demonstrated in cynomolgus monkey retina, to the interface between the ISs and OSs of both rods and cones (Figure 6D).

Subsequently, we examined samples obtained from the eye injected with dual AAVs encoding HA.PCDH15. Immunostaining against the HA tag demonstrated expression of exogenous PCDH15 within the photoreceptor layer in the inner/outer segment junction, which extended along OSs of cones and rods (Figure 6E). The localization was similar to the endogenous PCDH15 expression seen in the PBS-injected eye (Figure 6D). We quantified the transduction efficiency in all 3 bleb regions. Anti-HA labeling demonstrated high numbers of transduced cones and rods throughout bleb areas with approximately 94%–97% of cones labeled and approximately 96%–98% of rods (Figure 6F). No HA labeling was detected in the eye injected with vehicle (Figure 6G).

Altogether, the results show that the dual-AAV approach, effective in human retina organoids, was successfully translated to NHPs. High levels of exogenous full-length PCDH15 were reached in the monkey eye after subretinal injection (Figure 6, E and F).

## Discussion

Substantial progress has been achieved recently in the field of gene therapy for deafness (35, 36, 49–52) and blindness (14, 26, 53–55). This progress has usually involved gene addition therapy, which has proven effective for small genes with coding sequences that can be accommodated within a single AAV vector (<4.7 kb). However, certain genes, including many responsible for Usher syndrome, have coding sequences that exceed the capacity of single AAV vectors (1, 2, 56).

Here, we demonstrate the successful application of a dual-AAV vector strategy for gene addition therapy in auditory-vestibular and visual sensory systems using 4 different animal models: constitutive and conditional mouse models of USH1F deafness and balance dysfunction, cultured human retinal organoids, and intact NHP retina.

We employed a hybrid dual-AAV approach (31–34, 37, 39) to deliver the full-length PCDH15 coding sequence, which is too large to fit within a single AAV capsid, by using 2 AAV vectors (AAV-Pcdh15 5′ and AAV-Pcdh15 3′) containing different portions of the coding sequence. Upon transduction of target cells with both vectors, a highly recombinogenic AK sequence facilitated recombination of DNA from the 2 vectors, resulting in the production of full-length *PCDH15* mRNA and PCDH15 protein.

In this study, we selected the AAV9-PHP.B capsid for delivery and confirmed its excellent ability to transduce sensory cells within the mouse cochlea (43–45), as well as photoreceptors in both human retinal organoids and NHP retinas.

The dual-AAV approach was initially validated in vitro using HEK293 cells, confirming efficient recombination and splicing that led to PCDH15 protein production. In vivo rescue of hearing function was assessed in conditional knockout *Pcdh15^fl/fl^*, *Myo15a-Cre^+/–^* mice. In these mice, as previously described, normal hair cell morphology was maintained at the time of dual-AAV treatment and before deletion of endogenous *Pcdh15* by Cre recombinase (27). This allows normal hair cell development before deletion. The mRNA from dual-AAV delivery could substitute for the native mRNA, potentially without markedly altering the total protein output. This also allowed us to evaluate the full effect of the *Pcdh15* transgene delivered via dual AAV without complication from loss of the target cells. The conditional knockout *Pcdh15^fl/fl^*, *Myo15a-Cre^+/–^* mice, which suffered a profound hearing loss if left untreated, showed that dual-AAV delivery of *Pcdh15-CD2* (17, 40) robustly preserved auditory function, with some mice reaching control-level thresholds. This treatment also preserved stereocilia bundle morphology and mechanotransduction in hair cells. Immunofluorescence and immunogold scanning electron microscopy confirmed the precise trafficking and localization of exogenous PCDH15 at cochlear stereocilia tips. Treated control mice showed no signs of hearing-related toxicity.

Vestibular function was also assessed using a constitutive knockout *Pcdh15^–/–^* mouse model, which exhibited severe vestibular dysfunction. The dual-AAV treatment preserved vestibular function at near-normal levels in these mice, as indicated by improved performance in various behavioral tests. The *Pcdh15^–/–^* model represents a more severe form of USH1F compared with the *Pcdh15^fl/fl^*, *Myo15a-Cre^+/–^* model. While the *Pcdh15^–/–^* model showed marked preservation of balance, the deafness phenotype was not improved by the dual-AAV treatment in these mice. In *Pcdh15^–/–^* mice, despite the rescue of *Pcdh15* expression, the vector delivery apparently occurred too late to fully preserve the bundle architecture, leading to no ABR rescue. Our findings suggest that intervention beyond a critical period may not effectively preserve hearing. In contrast, vestibular hair cells may be more responsive to therapy, as they continue to differentiate even after birth in mice (57). Early intervention targeting vestibular hair cells produced full rescue of vestibular function. We hypothesize that the differential response between hearing and balance preservation is due to the specific timing of hair cell development relative to AAV administration. Vestibular hair cells, crucial for balance, might have a later developmental window, more amenable to the effects of AAV treatment (58) compared with that of cochlear hair cells involved in hearing. This finding has important therapeutic implications, highlighting the potential for differential treatment strategies tailored to the specific sensory deficits in USH1F.

The use of human retinal organoids derived from human induced pluripotent stem cells has enabled both the examination of retinal development and the study of retinal diseases (59–64). They consist of 3 nuclear layers and 2 synaptic layers, replicating the cellular organization observed in the adult retina, and they exhibit photoreceptor responsiveness to light stimuli and facilitate synaptic transmission of visual information, resulting in light responses in second- or third-order retinal cells (62). We therefore examined the photoreceptor ultrastructure within retinal organoids and the localization of PCDH15. The emergence of nascent calyceal processes at the apical ends of the ISs, where we found PCDH15, validates the suitability of organoids as a model for PCDH15 delivery. Immunofluorescence and immunogold scanning electron microscopy imaging confirmed the successful transduction and localization of HA-tagged PCDH15 in photoreceptors, particularly in the calyceal processes and ISs.

Old World monkeys are an appropriate model for investigating ocular diseases. Since only primates possess a macula, NHP models play a crucial role not just in uncovering the biological mechanisms behind high-acuity vision but also in advancing therapeutic development (65). Recent years have seen successful transitions from basic research to clinical trials and even the approval of the first treatments for inherited and age-related retinal dystrophies (66). In our study, subretinal injection of dual AAV in an NHP produced high levels of exogenous PCDH15 expression in both rods and cones, indicating the potential applicability of this approach to human patients.

In summary, the dual-AAV approach holds substantial promise for the development of gene replacement therapy for USH1F. The potential preservation of both auditory and vestibular functions indicates that the dual-AAV strategy could present a promising therapeutic option, especially considering that treatments for deafness, such as cochlear implants, are already available, whereas there are currently no treatments for vestibular dysfunction that can be offered to patients with USH1F. Furthermore, the successful application of the dual-AAV approach in human retinal organoids and NHPs provides a foundation for future translational studies aimed at treating retinal degeneration associated with USH1F. These findings provide a strong foundation for further research and the development of potential treatments for USH1F and related conditions.

## Methods

### Sex as a biological variable.

The study did not consider the sex of the mice and NHPs. Initial experiments showed no sex-specific hearing or vision differences, so both male and female animals were used in the experiments.

### Dual-PCDH15 study design.

The dual-PCDH15 strategy was based on the hybrid strategy (30, 31, 33). To promote recombination between the 5′ and 3′ viruses, we inserted the highly recombinogenic AK sequence derived from the F1 phage genome (32). The 5′ vector harbors a splice donor site upstream of the AK sequence, while the 3′ vector harbors a splice acceptor site immediately downstream of the AK sequence. The coding sequence of the mouse CD2-1 isoform of *Pcdh15* was used for the initial design (NM_001142742.1). Experiments in human retinal organoids and in the green monkey retina used the coding sequence for the human CD1-1 isoform (NM_001142763.2). The AAV expression cassettes tested in this study are shown in Figure 1A. We used an AAV transgene plasmid, flanked by AAV2 inverted terminal repeats. For all experiments, we used a 584 bp CMV promoter, which we had previously demonstrated to be effective in the cochlea (27). Additionally, 3′ viruses were engineered to include an IRES GFP element to facilitate the cotranslation of a fluorescent marker. These constructs also incorporated the woodchuck hepatitis virus post-transcriptional regulatory element (WPRE) and a bovine growth hormone (BGH) poly(A) sequence.

### Mouse models.

Animal handling and breeding were performed in the Harvard Medical School animal facility. All studies were performed on *Pcdh15^fl/fl^*, *Myo15a-Cre^+/–^* and *Pcdh15^R245X/R245X^* mice (here referred to as *Pcdh15^–/–^*), which were previously described (27, 28). The *Pcdh15^fl/fl^* mice lacking Cre recombinase displayed hearing sensitivity and bundle morphology comparable to those in wild-type mice (*Pcdh15^+/+^*). Therefore, in this study, *Pcdh15^fl/fl^ Cre^–^* mice were used as normal-hearing controls. All experiments were performed on *Pcdh15^fl/fl^*, *Myo15a-Cre* mice with a mixed C57BL/6J–129/Sv genetic background. The *Pcdh15^fl/fl^* and *Pcdh15^R245X/R245X^* mice were generated at Harvard Medical School, the *Myo15a-Cre* mice were provided by Christine Petit (Institut Pasteur, Paris, France) with assistance from Ronna Hertzano (University of Maryland School of Medicine, Baltimore, Maryland, USA), and the C57BL/6J mice were obtained from The Jackson Laboratory. Genotyping for the *Myo15a-Cre* mouse lines was conducted as previously described (46). *Pcdh15^–/+^* mice exhibited hearing sensitivity and bundle morphology similar to those in wild-type mice; thus, in this study *Pcdh15^–/+^* mice were used as normal controls for rescue experiments in *Pcdh15^–/–^* mice. All *Pcdh15^–/–^* mice were on a C57BL/6J genetic background. Genotyping for the *Myo15a-Cre* mouse lines was conducted as previously described (28).

### Viral vector production.

Most AAVs produced in this study were manufactured by the Viral Vector Core at Boston Children’s Hospital (Boston, Massachusetts, USA). Specifically, AAV9-PHP.B vectors were generated in HEK293 cells by polyethylenimine-mediated cotransfection of pAAV transfer plasmid, pHelper plasmid, and RepCap plasmid pUCmini-iCAP-PHP.B. One hundred twenty hours after transfection, both the medium and cells were collected. Subsequently, AAV9-PHP.B viruses were extracted and subjected to ultracentrifugation using a discontinuous density iodixanol (OptiPrep, Axis Shield) gradient. After ultracentrifugation, AAV vector–containing iodixanol fractions were isolated and concentrated via diafiltration. The purified AAV vectors were quantified using quantitative PCR, divided into single-use aliquots, and stored at –80°C until required, with thawing immediately before in vivo injections. For NHP experiments, AAVs were produced by PackGene Biotech.

### Transduction of HEK293 cells.

HEK293 cells (ATCC, CRL-1573) were plated on glass coverslips in DMEM with 10% FBS (Gibco) and penicillin/streptomycin (Pen/Strep; Invitrogen). On the following day, cells were transfected with Lipofectamine 3000 (Thermo Fisher Scientific) according to the manufacturer’s protocol. Plates were incubated at 37°C for 24 hours and then at 30°C for an additional 48 hours to promote high levels of protein expression.

HEK293 cells were transduced with AAV9-PHP.B vectors when they reached a low confluence of approximately 40%, using a multiplicity of infection of approximately 7 × 10^5^. The vectors were diluted in 200 μL of DMEM containing 1% FBS, penicillin, and streptomycin, and then applied directly onto coverslips to create a hydration bubble. These coverslips were then placed in a 37°C incubator. After 16–24 hours, the cells were washed with medium containing 1% FBS and 0.5× Pen/Strep and subsequently replenished with 2 mL of fresh medium. They were then cultured at 30°C for an additional 48 hours to promote high levels of protein expression. Cells were collected for mRNA or fixed for immunofluorescence.

### RNA extraction, cDNA production, reverse transcription, PCR amplification, and sequencing.

Total RNA was extracted from transduced HEK293 (ATCC, CRL-1573) cells using the Zymo Quick-RNA Microprep Kit (Zymo Research, R1050). Reverse transcription was performed with Invitrogen’s SuperScript IV VILO Master Mix with ezDNAse enzyme (Invitrogen, 11766050). *Pcdh15-CD2* cDNA was amplified with a forward primer that anneals to a sequence in the 5′ vector (5′-AGGATGAAAACGATCACCCCC-3′) and a reverse primer that anneals to a sequence in the 3′ vector (5′-GGTATGATGAGCCGGTAGGC-3′). The expected product size for the properly spliced *Pcdh15-CD2* cDNA splice junction site is 154 bp. The DNA Gel Extraction Kit (Monarch, T1020S) was used to gel-extract PCR products. Purified PCR products were subcloned, transformed into competent cells, mini-prepped, and Sanger-sequenced using the NEB PCR Cloning Kit (New England Biolabs, E1202S).

### Expression in human retinal organoids.

Wild-type human retina organoids were generated and maintained at the Institute of Molecular and Clinical Ophthalmology Basel as was described previously (62). These organoids were subsequently transduced with AAV vectors in a 96-well plate using the following dosages: dual AAV (AAV-HA.Pcdh15 5′ + AAV-Pcdh15 3′), 1.7 × 10^12^ VGC; AAV-HA.Pcdh15 5′, 8.5 × 10^11^ VGC; AAV-Pcdh15 3′, 8.5 × 10^11^ VGC; AAV-CMV-GFP, 8.5 × 10^11^ VGC. Transduced organoids were maintained in 50 μL of 3:1 N2 medium at 37°C in 5% CO_2_. The composition of 3:1 N2 medium included DMEM (Gibco, 10569-010) supplemented with 20% Ham’s F12 Nutrient mix (Gibco, 31765-027), 10% heat-inactivated FBS (Millipore, ES-009-b), 1% N2 Supplement (Gibco, 17502-048), 1% NEAA Solution (MilliporeSigma, M7145), 100 μM taurine (MilliporeSigma, T0625), and 1 μM retinoic acid (MilliporeSigma, R2625) (62). After 4 hours, 50 μL of medium was added to each well. One day later, 100 μL of medium was added to each well. After 24 hours and every 48 hours thereafter, the solution was completely exchanged with fresh medium. Five weeks later, samples were fixed with 4% formaldehyde.

### Expression in NHP retina.

For in vivo delivery in a species with greater relevance to humans, we chose an Old World primate, the green monkey (*Chlorocebus sabaeus*). Injections were carried out by Virscio Inc. Before injection, the animal underwent a thorough ophthalmological assessment conducted by a veterinary ophthalmologist, and baseline screening to assess AAV9 neutralizing antibody seronegativity. The animal received methylprednisolone (40 mg i.m.) weekly for 4 weeks, starting on the day prior to dosing. Anesthesia was achieved with intramuscular ketamine (8 mg/kg) and xylazine (1.6 mg/kg). Pupil dilation with accomplished using topical 10% phenylephrine, 1% tropicamide, and/or 1% cyclopentolate. After placement of the scleral ports, a contact vitrectomy lens was positioned on the cornea using 0.9% saline as a coupling agent. A 25-gauge light pipe was inserted through the left scleral port into the vitreous cavity for intraocular illumination. Simultaneously, a subretinal cannula was introduced through the second scleral port. The cannula was gently advanced to touch the retinal surface in a specific location. Once the retinal surface showed a slight blanching at the point of contact, the vector was administered through the cannula. Three blebs were introduced, in the superior, inferior, and temporal regions of the retina, each containing dual AAV vectors at a dosage of 2.5 × 10^12^ VGC per bleb (totaling 7.5 × 10^12^ per eye), with each bleb receiving 50 μL of virus. The second eye served as a control and received an injection of a formulation buffer consisting of PBS with 0.001% F-68, with the same distribution of 3 blebs in the superior, inferior, and temporal regions of the retina, each containing 50 μL.

### RNA extraction, cDNA production, and quantitative reverse transcription PCR in NHPs.

Three cynomolgus monkey (*Macaca fascicularis*) cadavers were acquired post-euthanasia from an unrelated study at Massachusetts General Hospital (Boston, Massachusetts, USA) and transferred to Harvard Medical School as approved by IRB Ex Vivo Animal Tissue Importation Amendment ID 14-111-A02. Cochlear and vestibular system tissue from 5 inner ears and retinal tissue from 6 eyes were extracted and transferred into Trizol solution (Invitrogen) surrounded by a liquid nitrogen bath. Once cochlear and retinal tissues were digested, RNA and DNA isolation was carried out per the Trizol manufacturer’s instructions. Approximately 1–5 μg of RNA was used to create cDNA using the SuperScript III First-Strand Synthesis System for reverse transcription PCR (RT-PCR) (Thermo Fisher Scientific, 18080051). Quantitative RT-PCR (RT-qPCR) was carried out with SYBR Select Master Mix (Thermo Fisher Scientific, 4472908) on an ABI StepOnePlus qPCR machine using the following primers: *PCDH15_CD1* forward primer 5′-CTCTATGAAGAACTTGGAGACAGCT-3′, reverse primer 5′-GGAAGAAAAGGGCATCACAACTTG-3′; *PCDH15_CD2* forward primer 5′-CTCTATGAAGAACTTGGAGACAGCT-3′, reverse primer 5′-CCTCACTAGGCTCTCTAATTTCAACTT-3′; *PCDH15_CD3* forward primer 5′-CTCTATGAAGAACTTGGAGACAGCT-3′, reverse primer 5′-CTCGATCTACAACTAACTTGATCATTCT-3′. Expression of *PCDH15-CD1* isoforms was measured via ΔΔCt of the most prominent isoform in comparison with *GAPDH* or *RPS19* calibrator genes.

### AAV round window membrane injection in neonatal mice.

P1 pups were anesthetized using cryoanesthesia and kept on an ice pack during the procedure. Injections were done through the round window membrane as previously described (43). Briefly, a small incision was made beneath the external ear. The round window niche was identified visually, and the viral vector solution was delivered via a micropipette needle at a controlled rate of 150 nL/min. The surgical incision was closed using two 7-0 Vicryl surgical sutures (Ethicon). After the injection, standard postoperative care protocols were implemented.

### ABR and DPOAE testing.

ABRs and DPOAEs were recorded following established procedures (27, 67), using a custom acoustic system developed by Massachusetts Eye and Ear. Adult mice aged 5 weeks were given anesthesia using a ketamine/xylazine mixture and were placed on a 37°C heating pad throughout the recording session. For ABR recordings, 3 subdermal needle electrodes were used. Tone-pip stimuli with a duration of 5 milliseconds and a rise-fall time of 0.5 milliseconds were delivered at frequencies ranging from 4.0 kHz to 32 kHz. Sound levels were increased in 5-dB increments, starting from approximately 20 dB sound pressure level (SPL) and increasing up to 80 dB. ABR Peak Analysis software (version 1.1.1.9, Massachusetts Eye and Ear) was used to determine ABR thresholds and measure peak amplitudes. DPOAEs were recorded for primary tones with a frequency ratio of f2/f1 = 1.2, where L1 was set as L2 + 10 dB. The f2 frequency ranged from 5.6 kHz to 32 kHz in half-octave increments. Primary tone levels were adjusted in 5-dB increments, spanning from 10 dB SPL to 70 dB SPL for f2.

### Open field test.

We used a square 37 × 37 cm^2^ arena with uniform, low-level illumination for our experiments. The testing took place when the animals were age P35. Each animal was positioned at the side of the arena, and position was recorded with video for a duration of 4 minutes. To prevent any potential olfactory distractions, the arena was thoroughly cleaned between test sessions. Video footage was subsequently analyzed using ImageJ software (NIH), and open field path outlines were generated. During the 4-minute observation period, we quantified the number of full-circle rotations, including both clockwise and counterclockwise turns.

### Rotarod and swimming tests.

The rotarod test was conducted over 2 days. On the first day, mice were positioned within an enclosed housing on a rotating rod, initially spinning at a constant rate of 4 revolutions per minute (rpm) for 5 minutes. Mice that fell during this training session were promptly placed back on the rotating rod. On the second day, the trained mice were once again placed on the spinning rod, but this time with a start speed of 4 rpm and acceleration rate of 20 rpm/min. The time each animal managed to remain on the device before falling to the floor of the housing was monitored by a timer and recorded after each trial. A 5-minute resting interval was enforced between trials, and a total of 5 trials were conducted for each mouse. The latency to fall off the rotarod was recorded. In the swimming test, the mice were placed in a tank filled with water, which forced them to swim. The time mice could swim before needing rescue was recorded.

### FM1-43 loading in adult cochlea.

Adult mice were subjected to anesthesia using isoflurane through an open drop method and were subsequently euthanized by cervical dislocation followed by decapitation. The otic capsules from the mice were carefully extracted and then placed in Leibovitz’s L-15 medium (Gibco). Under a stereomicroscope, the apical and mid-apical regions of the cochlea were microdissected. The tectorial membrane was gently pulled away to expose the sensory epithelium. A solution of FM1-43 (2 μM in L-15) was directly applied to the exposed epithelium at room temperature and left on for 1 minute. Subsequently, a solution of SCAS (0.2 mM) was applied. Imaging of the organs of Corti was carried out using an Olympus upright FV1000 confocal microscope equipped with a ×60 1.1-NA water-dipping objective lens.

### Immunofluorescence labeling of mouse cochleae and HEK293 cells.

Adult mice were anesthetized, then humanely euthanized by cervical dislocation followed by decapitation. The cochleae were dissected and fixed with 4% formaldehyde in HBSS for 1 hour at room temperature. Afterward, they were decalcified in 10% EDTA for 2 days. Once decalcified, the organs of Corti were microdissected and blocked with 10% donkey serum. Subsequently, the samples were stained with an anti-HA antibody (Abcam ChIP Grade, ab9110) diluted to 1:500 in 10% donkey serum and incubated overnight, followed by multiple rinses in HBSS. The samples were then incubated in a blocking solution (10% donkey serum) for 30 minutes at room temperature. After that, they were incubated overnight at room temperature with a donkey anti-rabbit IgG secondary antibody conjugated to Alexa Fluor 594 (Invitrogen, R37119), diluted to 1:500 in the blocking solution, which also included Alexa Fluor 405 phalloidin (Invitrogen, A30104) diluted to 1:20 to label actin. After the secondary antibody steps, the samples underwent several rinses and were mounted on Colorfrost glass slides (Fisher Scientific) using ProLong Gold Antifade mounting medium (Thermo Fisher Scientific).

The imaging was conducted using a Nikon Ti2 inverted spinning disk confocal microscope with Nikon Elements Acquisition Software AR 5.02, using the following objectives: a Plan Apo λ ×100/1.45 oil, a Plan Fluor λ ×40/1.3 oil, and a Plan Apo λ ×60/1.4 oil.

For immunocytochemistry in HEK293 cells, transfected cells were fixed with 4% formaldehyde for 1 hour, then washed 3 times with HBSS, and subsequently blocked with 10% donkey serum. We used either a sheep polyclonal anti-PCDH15 antibody (R&D Systems, AF6729) or a rabbit anti-HA (C29F4) antibody (Cell Signaling Technology, 3724), both diluted to 1:200 in 10% donkey serum, and incubated samples for 24 hours at room temperature, followed by several rinses in HBSS. Next, the samples were incubated in a blocking solution for 30 minutes and then incubated overnight at room temperature with a donkey anti-sheep IgG secondary antibody conjugated to Alexa Fluor 594 (Invitrogen, A-11016) or donkey anti-rabbit immunoglobulin IgG secondary antibody conjugated to Alexa Fluor 594 (Invitrogen, R37119) diluted to 1:500 in the blocking solution. After the secondary antibody incubation, the samples underwent several rinses in HBSS and were mounted on Colorfrost glass slides using ProLong Gold Antifade mounting medium. Imaging was carried out using an Olympus FluoView 1000 confocal microscope equipped with a ×60/1.42-NA oil-immersion objective.

### Immunofluorescence labeling of NHP retina and human retina organoids.

NHPs were euthanized and perfused with heparinized saline followed by 4% formaldehyde. Collected eye globes were postfixed for another 24 hours in 4% formaldehyde before the retinas were dissected. Human retinal organoids were fixed with 4% formaldehyde followed by a triple wash with PBS. Next, retina samples and organoids were cryoprotected by incubation in gradient concentrations of sucrose and then embedded in OCT compound and stored at −80°C before sectioning. Cryosections were generated using a Leica CM 3050 S cryostat at 30 μm step size.

For immunofluorescence labeling, the following primary antibodies and secondary antibodies were used: mouse anti-HA antibody (1:200) (BioLegend, 16B12), rabbit anti-HA antibody (1:200) (Abcam, ab9110), rabbit anti-ARR3 antibody (1:200) (Sigma Aldrich, HPA063129), sheep polyclonal anti-PCDH15 (1:200) (R&D Systems, AF6729), mouse monoclonal anti-rhodopsin (1:500) (MilliporeSigma, MAB5316), donkey anti-rabbit IgG secondary antibody conjugated to Alexa Fluor 594 (1:200) (Invitrogen, R37119), donkey anti-sheep IgG conjugated to Alexa Fluor 488 (1:200) (Invitrogen, A-11015), donkey anti-mouse IgG conjugated to Alexa Fluor 488 (1:200) (Invitrogen, A32766), donkey anti-mouse IgG conjugated to Alexa Fluor 405 (1:200) (Invitrogen, A48257), and donkey anti-rabbit IgG conjugated to Alexa Fluor 488 (1:200) (Invitrogen, A-21206). Samples were blocked with 10% donkey serum for 1 hour at room temperature. Antibodies were diluted in 10% donkey serum and incubated overnight at room temperature, followed by several rinses in HBSS. Next, samples were incubated in a blocking solution for 30 minutes and incubated overnight at room temperature with a secondary antibody in the blocking solution. We used DAPI (Invitrogen, D1306) or Hoechst 34580 dye (Invitrogen, H21486) to label cell nuclei (1:500) and BODIPY (4,4-difluoro-4-bora-3a,4a-diaza-*s*-indacene; Invitrogen, D3835) to label membranes (1:1500). Imaging was performed with a Nikon Ti2 inverted spinning disk confocal microscope.

### Quantification of confocal microscopy data.

Microscopy data analysis and quantification were done in the Fiji distribution of ImageJ v1.53. Transduction efficiency in cells was evaluated as previously described (27). Hair cells were identified with phalloidin staining of bundles and photoreceptors identified with BODIPY, and transduced cells identified by positive HA-tag labeling. Control samples without AAV were used to correct for autofluorescence. Segments with dissection-related damage were removed from the analysis.

GraphPad Prism 7 software was used to generate the graphs and perform the statistical analysis. The results are shown as mean ± SEM or mean ± SD as indicated in figure legends. Randomization was used whenever possible.

### Conventional scanning electron microscopy.

Scanning electron microscopy in adult mice, NHP retina, or retina organoids was performed as previously described (27, 68). Immediately after extraction, cochleae underwent prefixation by immersion in a solution containing 1% glutaraldehyde and 4% formaldehyde, both in 0.1 M cacodylate buffer (pH 7.2) supplemented with 2 mM CaCl_2_, for 1 hour at room temperature. After the prefixation step, the samples were postfixed using 2.5% glutaraldehyde in 0.1 M cacodylate buffer (pH 7.2), supplemented with 2 mM CaCl_2_, for an additional hour at room temperature. They were next rinsed in 0.1 M cacodylate buffer (pH 7.2) and then with distilled water. The cochlear bone was removed using a 27-gauge needle, followed by microdissection of the organ of Corti.

Next, the samples were immersed in a saturated aqueous solution containing 1% osmium tetroxide for an hour in a light-controlled environment, and then were postfixed using a 1% tannic acid aqueous solution for an hour in the dark. Finally, the samples underwent a series of rinsing, dehydration, and critical-point drying. The prepared samples were mounted on aluminum stubs equipped with carbon-conductive tabs. They were then sputter-coated with a platinum layer to a thickness of 5 nm using an EM ACE600 sputter coater (Leica) and imaged with a Hitachi S-4700 field-emission scanning electron microscope equipped with a backscattered electron detector.

### Immunogold scanning electron microscopy in mouse cochlea and human retina organoids.

Immunogold scanning electron microscopy was conducted in accordance with previously described protocols (27, 68). Cochleae and human retina organoids were fixed using 4% formaldehyde. After fixation and washing, samples were subjected to a 2-hour blocking step at room temperature using 10% normal goat serum. Next, the samples were incubated with primary antibodies for 24 hours at room temperature and were subsequently rinsed in HBSS. An anti–HA tag antibody (Abcam, ab9110) at a 1:200 dilution in 10% donkey serum was used to label the HA tag. After rinsing, the samples were blocked for 30 minutes at room temperature using 10% normal goat serum. They were then incubated overnight at room temperature with a secondary antibody solution consisting of 12 nm Colloidal Gold AffiniPure Goat Anti-Rabbit IgG (Jackson ImmunoResearch, 111-205-144) at a 1:30 dilution in the blocking solution. After the application of the secondary antibodies, the samples underwent rinses in HBSS.

Finally, the samples were prepared for observation. This involved a dehydration step, followed by critical-point drying, mounting, and sputter-coating with palladium in the range of 3–5 nm. Samples were imaged using a Hitachi S-4700 scanning electron microscope.

### Statistics.

All experiments, except for the ones noted below, were replicated in at least 3 independent experiments using separate samples, such as mice, organoids, or plates with HEK cells.

In NHP experiments, an initial detailed examination of PCDH15 expression and photoreceptor structure was conducted using immunofluorescence and scanning electron microscopy on 1 cynomolgus monkey, with 4 samples taken from different areas of the retina. Evaluation of dual-AAV delivery in retina was conducted in 1 green monkey, in both eyes. Three blebs were introduced in each eye, in the superior, inferior, and temporal regions of the retina. Quantification was performed on at least 5 regions from each bleb. For experiments involving RT-qPCR, 5 whole inner ears or the retinas from 6 eyes were used.

Only samples displaying notable dissection-related damage were excluded from the analysis. No other data were omitted. All figures were created using Adobe Illustrator 2024 (v28.5). The ABR data were organized using Microsoft Excel 2016 (v16.0.5378.1000, Microsoft). Graphs were generated and statistical analysis was conducted using Prism 10 software (v10.02.3, GraphPad Software Inc.). Statistical significance was assessed using 1-way ANOVA followed by Dunnett’s test for the behavioral vestibular tests, and 1-way ANOVA followed by post hoc Tukey’s test to reveal tissue-specific *PCDH15* isoform expression. A *P* value less than 0.05 was considered statistically significant. The results are presented as mean ± SEM as specified in the figure legends. Randomization was employed whenever possible.

### Study approval.

All mouse experiments were carried out in accordance with ethical guidelines under protocol IS00001452, approved by the Institutional Animal Care and Use Committee at Harvard Medical School (Boston, Massachusetts, USA). These studies adhered to NIH guidelines.

### Data availability.

All data generated or analyzed during this study are included in this article and its supplemental information files. Values for all data points in graphs are reported in the Supporting Data Values file.

## Author contributions

MVI conceptualized the study; acquired, analyzed, and interpreted data; visualized in vivo and in vitro experiments; and wrote the manuscript. DMH performed plasmid cloning; acquired data; analyzed and interpreted data of ABR experiments, RT-PCR experiments, and some in vitro experiments; and wrote the manuscript. EMM performed plasmid cloning; acquired data; and analyzed and interpreted data of in vitro experiments. KTB performed RT-PCR experiments; analyzed disease-associated variants; and wrote the manuscript. MW acquired data and analyzed data of human retina organoid experiments. XC acquired data and analyzed data of NHP experiments. AJK acquired data of ABR experiments. YL performed neonatal injections. CWP generated R245X mice and performed RT-PCR experiments in NHPs. BG supervised the study; interpreted data of human retina organoid experiments; and wrote the manuscript. DPC conceptualized and supervised the study; interpreted data; and wrote the manuscript.

## Supplementary Material

Supplemental data

Unedited blot and gel images

Supporting data values

## Version 1 10/23/2024

In-Press Preview

## Version 2 12/02/2024

Electronic publication
