# Supplementary material for: PCDH15 dual-AAV gene therapy for deafness and blindness in Usher syndrome type 1F models
Source: J Clin Invest. 2024 Oct 23;134(23):e177700. doi: 10.1172/JCI177700 (PMC11601915; doi:10.1172/JCI177700)
Supplement: Supplemental data [file jci-134-177700-s018.pdf]

Supplementary Information for

PCDH15 Dual-AAV Gene Therapy for Deafness and Blindness in Usher Syndrome Type 1F Models

Maryna V. Ivanchenko[1], Daniel M. Hathaway[1], Eric M. Mulhall[1], Kevin T. Booth[1], Mantian Wang[2,3], Cole W. Peters[1], Alex J. Klein[1], Xinlan Chen[1], Yaqiao Li[1], Bence György[2,3], David P. Corey[1]

1. Department of Neurobiology, Harvard Medical School, Boston, Massachusetts, USA

2. Institute of Molecular and Clinical Ophthalmology Basel, Basel, Switzerland

3. Department of Ophthalmology, University of Basel, Basel, Switzerland

Corresponding author

David P. Corey (email: david_corey@hms.harvard.edu)

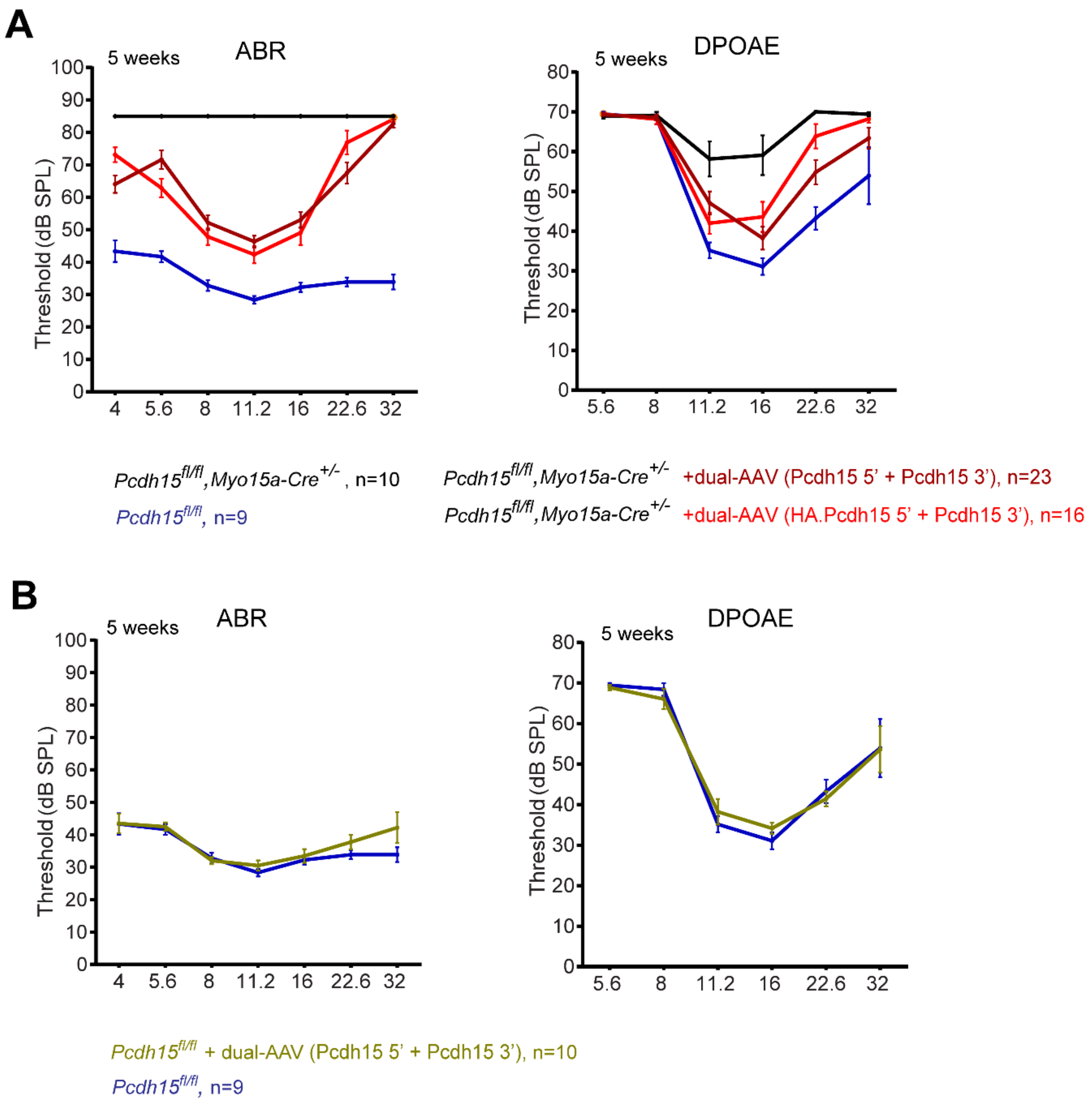

Supplementary Figure 1. (**A**) ABR and DPOAE recording in *Pcdh15*$^{fl/fl}$, *Myo15a-Cre*$^{+/-}$ mice injected at P1 with dual AAV vectors encoding either PCDH15 or HA.PCDH15 protein. Untreated *Pcdh15*$^{fl/fl}$, *Myo15a-Cre*$^{+/-}$ knockout mice had no ABR to 80 dB tones (*n*=10). Untreated *Pcdh15*$^{fl/fl}$ control mice had normal thresholds (*n*=9). The thresholds in *Pcdh15*$^{fl/fl}$, *Myo15a-Cre*$^{+/-}$ mice treated with untagged PCDH15 (*n*=23) or HA-tagged HA.PCDH15 (*n*=16) were the same, confirming that the HA tag does not impair function. (**B**) ABR and DPOAE recording in *Pcdh15*$^{fl/fl}$ hearing control mice injected with dual-AAVs encoding PCDH15 (*n*=10) demonstrated normal hearing at all frequencies, similar to uninjected *Pcdh15*$^{fl/fl}$ control mice (*n*=9), indicating no vector toxicity. Data are presented as mean ± SEM.

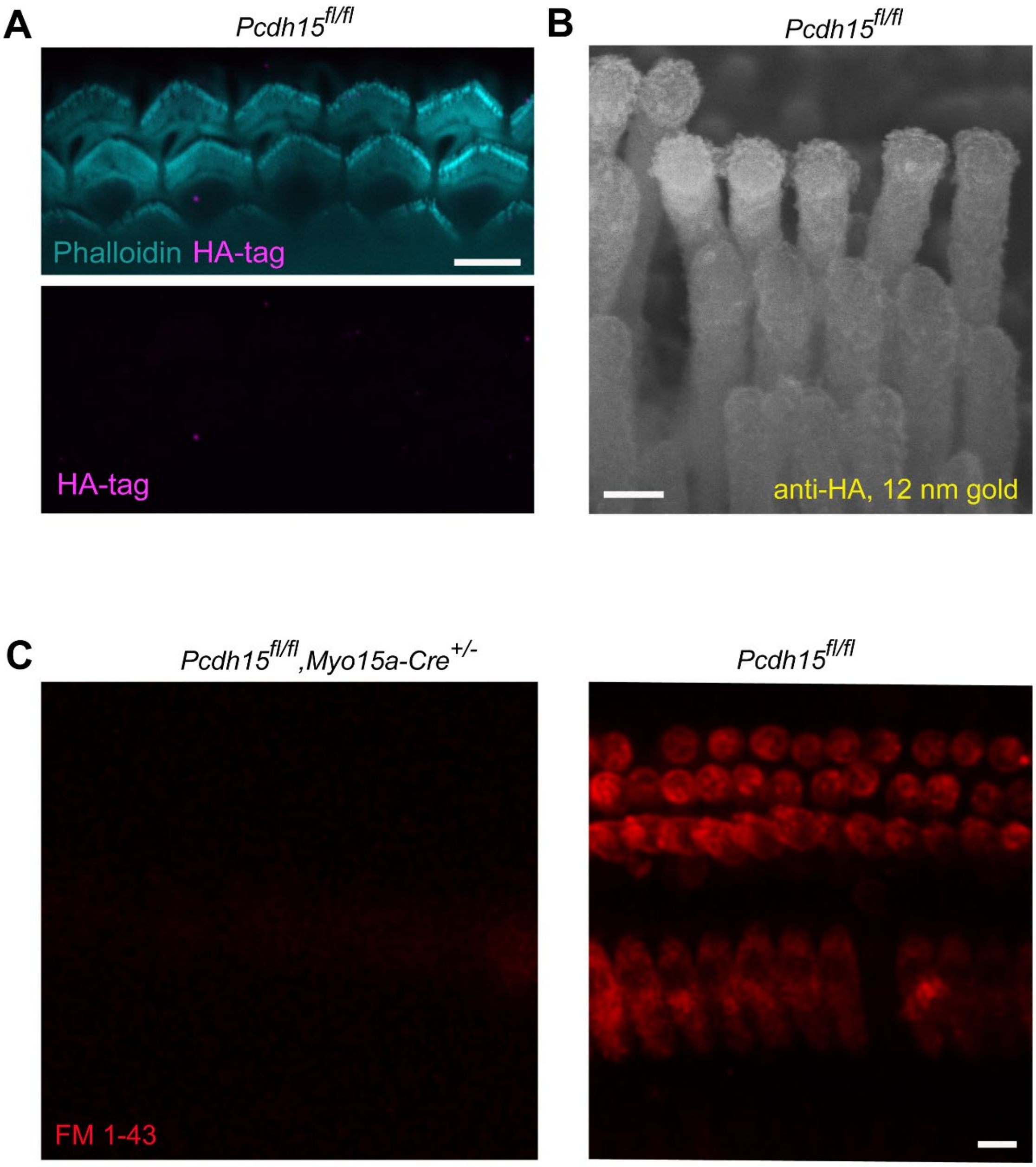

Supplementary Figure 2. Antibody validation and FM1-43 control. (**A**) Fluorescence microscopy of a 5-week-old uninjected *Pcdh15*$^{fl/fl}$ control cochlea stained with an anti-HA primary antibody and a fluorescently tagged secondary antibody. No fluorescent signal was observed, confirming the specificity of the antibodies (*n*=3). (**B**) Scanning electron microscopy of a 5-week-old uninjected *Pcdh15*$^{fl/fl}$ cochlea (*n*=3) immunostained with an anti-HA primary antibody and a 12 nm gold-conjugated secondary antibody. No gold beads were detected, confirming the specificity of the antibodies. (**C**) Representative confocal microscopy images of FM1-43 dye loading of IHCs and OHCs from the apical/mid-apical region of a cochlea at 5 weeks. *Left*, *Pcdh15*$^{fl/fl}$, *Myo15a-Cre*$^{+/-}$ knockout hair cells showed no FM1-43 label (*n*=4). *Right*, *Pcdh15*$^{fl/fl}$, *Cre-* control hair cells showed robust label (*n*=4). FM1-43 loading required the presence of PCDH15. Scale bars: (**A, C**) 5 µm, (**B**) 100 nm.

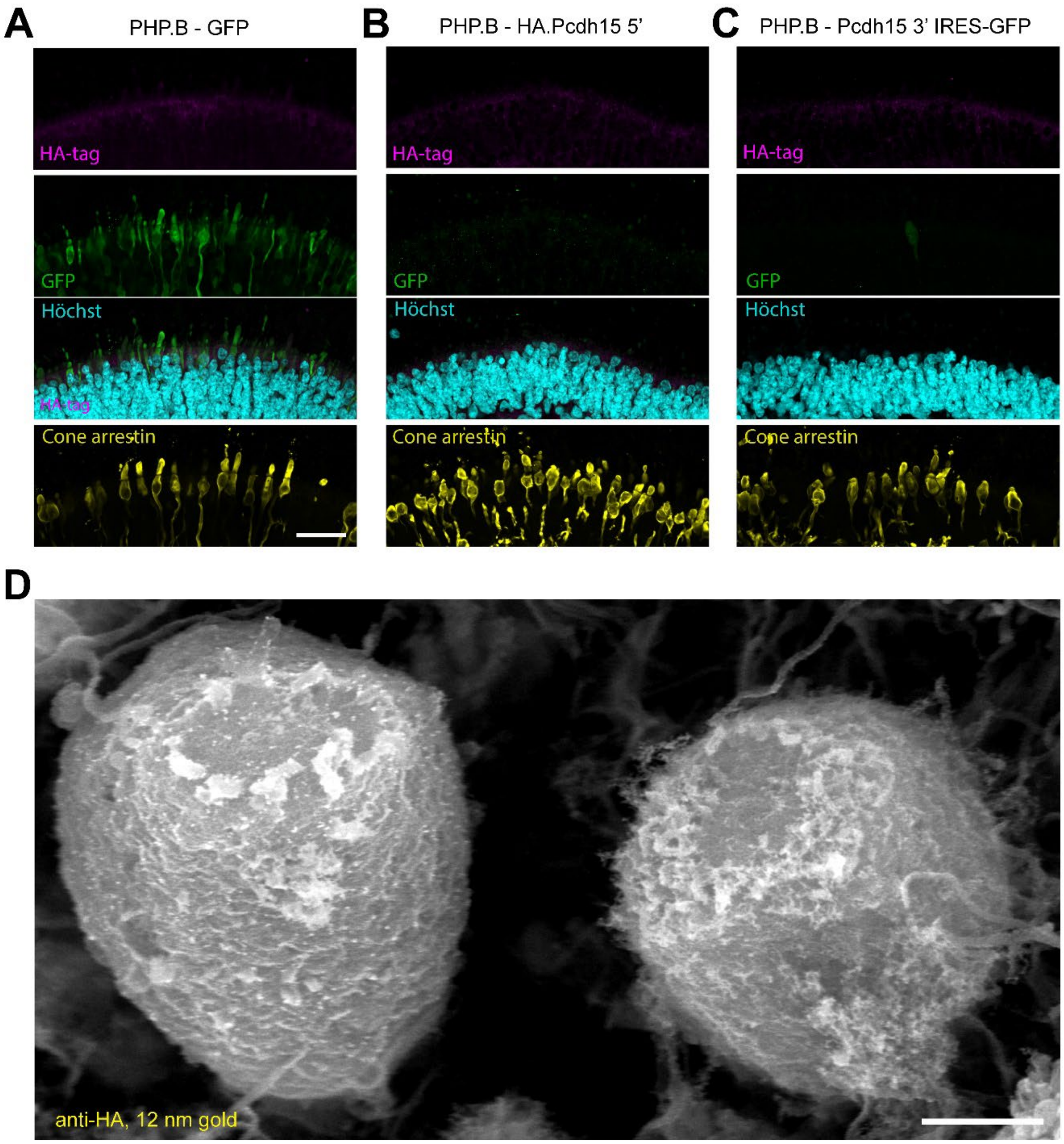

Supplementary Figure 3. Dual-AAV control and anti-HA antibody control. Transduction with control vectors in human retinal organoids. (**A**) Delivery of the AAV9-PHP.B-CMV-GFP vector encoding only GFP robustly transduced photoreceptors of retinal organoids and produced no anti-HA label (*n*=3). (**B**, **C**) The HA tag was also not detected when organoids were transduced with either the AAV9-PHP.B-HA.Pcdh15 5′ N-terminal vector or AAV9-PHP.B-Pcdh15 3′ C-terminal vector alone (*n*=3). (**D**) Scanning electron micrograph of two photoreceptors in a human retinal organoid, labeled with an anti-HA primary antibody and a 12-nm gold-conjugated secondary antibody (*n*=3). Many gold beads (white dots) were detected on the surface of the left photoreceptor, and were localized close to the nascent calyceal processes and inner segments. The right photoreceptor was unlabeled, suggesting that it was not transduced with both vectors. Lack of label on the right photoreceptor indicates that anti-HA label is not nonspecific. The left photoreceptor in this panel is shown again in Figure 5F of the main text. Scale bars: (**A**-**C**) 30 μm, (**D**) 1 μm.
