# Supplementary figures and images for: PCDH15 dual-AAV gene therapy for deafness and blindness in Usher syndrome type 1F models
Source: J Clin Invest. 2024 Oct 23;134(23):e177700. doi: 10.1172/JCI177700 (PMC11601915; doi:10.1172/JCI177700)

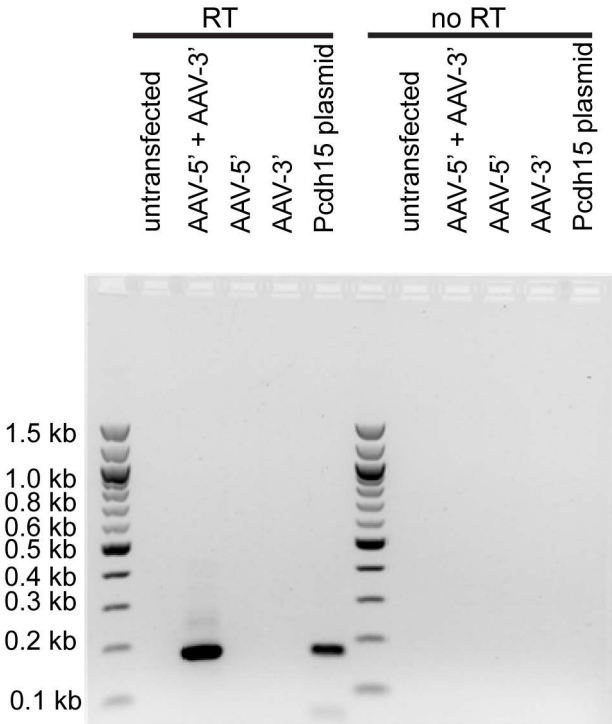

Full unedited gel for Figure 1B

Supplement: Unedited blot and gel images [file jci-134-177700-s019.pdf]
